# Role of novel protein acylation modifications in sepsis

**DOI:** 10.3389/fimmu.2025.1657194

**Published:** 2025-10-02

**Authors:** Jing Wang, Aifeng He, Lin Song, Wei Jiang, Lu Xu, Ruiqiang Zheng, Jiangquan Yu

**Affiliations:** ^1^ Northern Jiangsu People’s Hospital Affiliated to Yangzhou University, Yangzhou, China; ^2^ Intensive Care Unit, Northern Jiangsu People’s Hospital, Yangzhou, China; ^3^ Binhai County People’s Hospital, Yancheng, Jiangsu, China; ^4^ The Yangzhou Clinical College of Xuzhou Medical University, Xuzhou, Jiangsu Province, China

**Keywords:** sepsis, epigenetics, acylation, organ dysfunction, inflammation

## Abstract

Sepsis is a life-threatening organ dysfunction caused by a dysregulated host response to infection, exhibiting high global morbidity and mortality. Accumulating evidence indicates that post-translational modifications (PTMs), as pivotal epigenetic mechanisms, play a crucial role in regulating diverse biological processes. The significance of PTMs in sepsis is increasingly recognized, as they may influence disease progression by modulating protein stability, activity, and localization. In recent years, advances in mass spectrometry have elucidated a series of novel PTMs, including succinylation (Ksucc), S-palmitoylation, lactylation (Kla), crotonylation (Kcr), 2-hydroxyisobutyrylation (Khib), β-hydroxybutyrylation (Kbhb), and malonylation (Kmal). This review presents the first comprehensive analysis of the characteristics, functions, and implications of these seven lysine acylation modifications in the pathogenesis and progression of sepsis, aiming to provide valuable insights for diagnosis and therapeutic intervention.

## Introduction

Sepsis is a life-threatening organ dysfunction caused by a dysregulated host response to infection (1). In recent years, with the timely application of fluid resuscitation, antibiotic therapy, and organ function support, the mortality rate of sepsis has gradually declined. However, its high treatment costs remain a significant global public health concern (2, 3). The pathophysiology of sepsis is complex and frequently involves multiple organ dysfunctions, such as septic cardiomyopathy, acute liver injury, acute kidney injury, acute lung injury, coagulopathy, and endothelial dysfunction (4), Currently, effective diagnostic and therapeutic strategies to improve the prognosis of sepsis and reduce associated treatment costs remain lacking.

Epigenetics is a fundamental area of biology, encompassing DNA methylation, post-translational modifications, and non-coding RNAs, all of which contribute to heritable changes in biological phenotypes without altering the underlying genetic sequence (5, 6). Post-translational modifications (PTMs) are a central mechanism of epigenetic regulation and have attracted widespread attention in recent years due to their strong association with the pathogenesis and progression of various diseases, as well as their potential as therapeutic targets (7). PTMs refer to the chemical alterations of specific amino acid residues through covalent attachment of functional groups, which regulate protein structure and function. These modifications are ubiquitous in mammalian cells and play pivotal roles in modulating cellular molecular functions (8, 9). To date, over 400 types of PTMs have been identified to influence protein functionality. Among these, metabolic PTMs, particularly various newly discovered acylation modifications of histones or non-histone proteins, have been demonstrated to significantly impact the pathogenesis and progression of multiple diseases, including inflammatory, cancer, cardiovascular diseases, kidney diseases, and metabolic syndromes (7, 10). Acetylation was the first identified homogenous lysine modification linked to multiple cellular functions. In recent years, advancements in mass spectrometry, proteomics, and bioinformatics have facilitated the discovery of novel lysine modifications, including succinylation (Ksucc), S-palmitoylation, lactylation (Kla), crotonylation (Kcr), 2-hydroxyisobutyrylation (Khib), β-hydroxybutyrylation (Kbhb), and malonylation (Kmal). These modifications have emerged as significant areas of research in the field (6). The formation of certain novel PTMs requires specific energy metabolism intermediates as acyl donors (11). Accumulating evidence has revealed that acylation modifications constitute a dynamic and diverse network of metabolic modifications (10), whose dysregulation is closely associated with the pathogenesis of sepsis and its associated multi-organ dysfunction.

**Figure 1:**
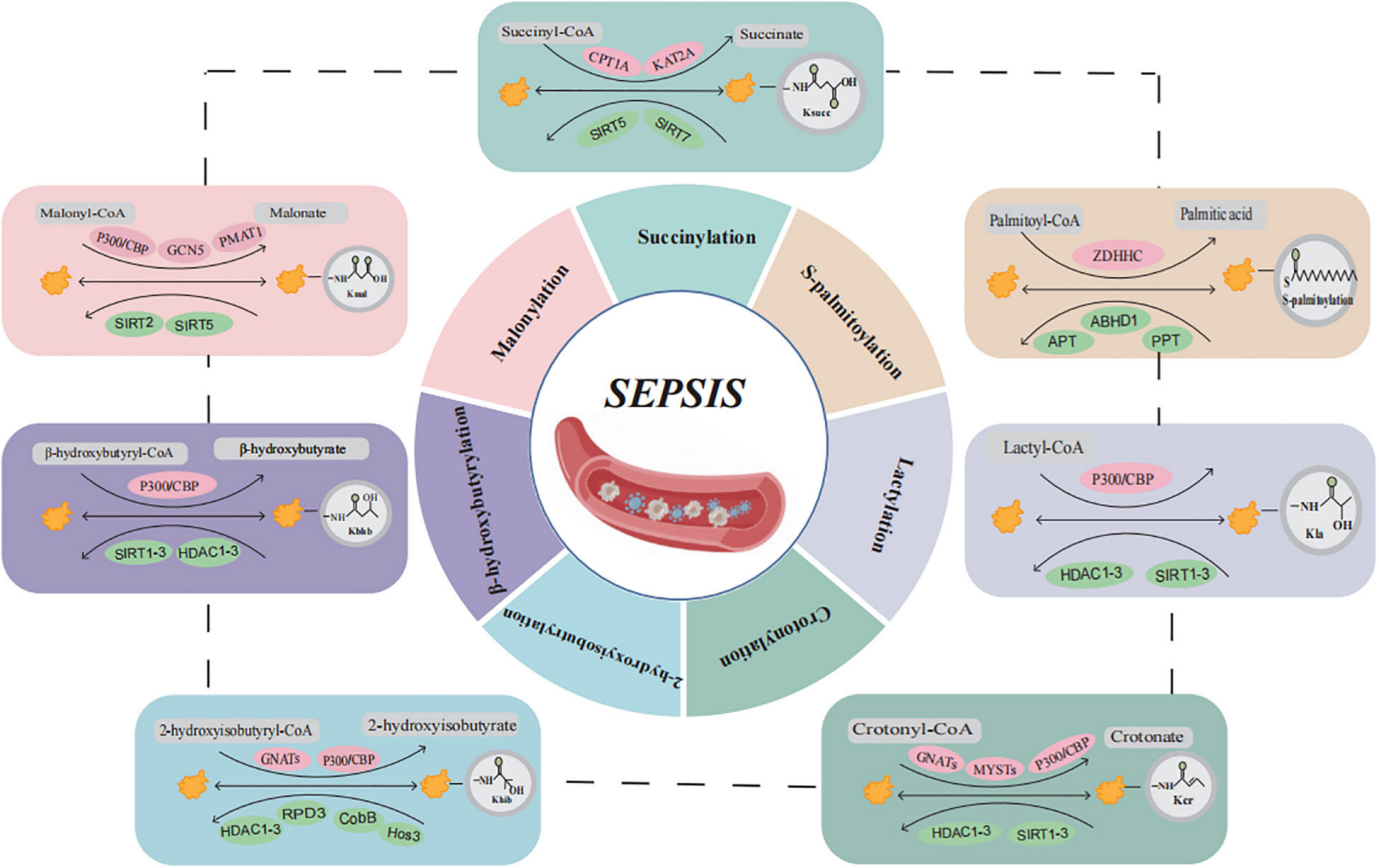
Overview of the composition and regulatory mechanisms of protein acylation in sepsis. Sepsis and multiple organ dysfunction are caused by protein acylation after translation for succinylation, S-palmitoylation, lactylation, crotonylation, 2-hydroxyisobutyrylation, β-hydroxybutyrylation, and malonylation may participate in the occurrence of sepsis development but have not yet been confirmed. (Created with BioGDP.com). CoA, coenzyme A; CPT1A, carnitine palmitoyl transferase 1A; KAT2A, lysine acetyltransferase 2A; SIRT5, sirtuin 5; SIRT7, sirtuin 7; ZDHHC, zinc finger Asp-His-His-Cys motif-containing; APT, acyl-protein thioesterase; PPT, Palmitoyl protein thioesterase; CBP, CREB binding protein; HDAC, histone deacetylases; SIRT1-3, sirtuin 1-3; GNATs, GCN5-related nacetyltrans-ferases; PMAT1, Plasma membrane monoamine transporter 1.

This review aims to summarize recent advancements in the study of novel acylation modifications in sepsis, with an emphasis on their molecular mechanisms and contributions to the pathogenesis of sepsis (**Figure 1**, **Table 1**). Through comprehensive analyses of Ksucc, S-palmitoylation, Kla, Kcr, Khib, Kbhb, and Kmal, we seek to offer new insights for future basic research and clinical applications, ultimately contributing to the development of diagnostic and therapeutic strategies for sepsis and its associated multi-organ dysfunction.

**Table 1:** Role of lysine acylation in sepsis.

PTMs	Acyl donor	Writers	Erasers	Disease	Mechanism	Reference
Succinylation	Succinyl-CoA	CPT1A,KAT2A,p300/CBP	SIRT5,SIRT7	SAKI,SCM,SALI	Chicoric Acid Inactivates the NLRP3 Inflammasome by Inhibiting the KAT2A/α-Tubulin Complex and Reducing α-Tubulin Acetylation; SIRT5-Mediated HOXA5 Desuccinylation Suppresses LPS-Induced Ferroptosis via FSP1 Activation	(20, 21, 30)
S-palmitoylation	Palmitoyl-CoA	ZDHHC family	APT,PPT	SCM,SILI	Vaccarin Inactivates the NLRP3 Inflammasome by Promoting Its Palmitoylation via zDHHC12; FASN Attenuates Sepsis-Induced Liver Injury by Inhibiting STING Palmitoylation via Malonyl-CoA	(49, 61)
Lactylation	Lactyl CoA	p300/CBP	HDAC1-3, SIRT1-3	SAKI	Lactate accumulation-mediated HMGB1 lactylation induces and exacerbates sepsis-associated acute kidney injury in mice via activation of the HMGB1-NETs signaling pathway; Increased lactate levels exacerbate SAKI through the lactylation of Fis1	(74, 75, 76)
Crotonylation	Crotonyl-CoA	p300/CBP, GNATs	SIRT1-3	sepsis	Knockdown of ACSS2, an enzyme involved in crotonyl-CoA synthesis, reduces the expression of inflammatory genes in LPS-stimulated RAW264.7 cells	(80)
2-hydroxyisobutyrylation	2-HydroxyisobutyrylCoA	Tip60,p300/CBP	HDAC1-3, SIRT3	sepsis	Khib is involved in the PI3K-Akt pathway, and may participate in sepsis	(87)
β-hydroxybutyrylation	β-hydroxybutyryl-CoA	P300/CBP	HDAC1-3, SIRT1-3	sepsis	β-Hydroxybutyrate functions as a histone deacetylase inhibitor to exert antioxidant effects and attenuate proinflammatory macrophage activation	(99, 100)
Malonylation	Malonyl-CoA	P300/CBP,GCN5(KAT2A)	SIRT5	sepsis	Lysine 213 of GAPDH undergoes Kmal modification, which enhances its enzymatic activity, dissociates it from TNF-α mRNA, and promotes the translation of TNF-α;	(108)

CoA, coenzyme A; CPT1A, carnitine palmitoyl transferase 1A; KAT2A, lysine acetyltransferase 2A; SIRT5, sirtuin 5; SIRT7, sirtuin 7; SAKI, sepsis acute kidney injury; SCM, Septic Cardiomyopathy; SALI, sepsis acute lung injury; NLRP3, NOD-like receptor family pyrin domain-containing 3; HOXA5, Homeobox A5; FSP1, ferroptosis suppressor protein 1; APT, acyl-protein thioesterase; PPT, Palmitoyl protein thioesterase; SILI, sepsis-induced liver injury; HDAC1-3, histone deacetylase 1-3; HMGB1, High mobility group box 1; GNATs, GCN5-related nacetyltrans-ferases; ACSS2, acyl-CoA synthetase short-chain family member 2; GCN5(KAT2A), acetyltransferase 2A.

## Succinylation (Ksucc)

Ksucc is a PTM involved in the partial transfer of succinyl coenzyme A (CoA) to lysine residues (ϵ-amino), and can be produced by elevated levels of succinic acid. This post-translational modification involves the enzymatic addition of succinyl groups to specific amino acid residues in proteins, playing a critical role in regulating innate immunity and inflammatory responses (**Figure 1**, **Table 1**) (12, 13). Acylation reactions are catalyzed through both enzymatic and non-enzymatic mechanisms. In enzymatic processes, acyltransferases and deacylases play a key role in the transfer and removal of acyl groups (6). Dysregulation of succinylation modification can lead to alterations in the activity and function of proteins involved in energy metabolism and downstream epigenetic modifications, which are closely associated with the pathogenesis and progression of various diseases, including inflammation and cancer (14, 15). Succinyl-CoA, derived from the tricarboxylic acid (TCA) cycle, serves as the primary substrate for Ksucc (16). Ksucc represents a reversible, dynamic, abundant, and evolutionarily conserved histone modification in eukaryotic cells, with identified modification sites present on histones H2A, H2B, H3, and H4 (17). The Ksucc modification is dynamically regulated by both succinyltransferases, such as carnitine palmitoyltransferase 1A (CPT1A) and lysine acetyltransferase 2A (KAT2A), as well as desuccinylases, including sirtuin 5 (SIRT5) and sirtuin 7 (SIRT7) (18, 19). KAT2A (also known as GCN5) was originally identified as a histone acetyltransferase. Research has indicated that chicoric acid inhibits the KAT2A/α-tubulin complex, leading to reduced acetylation of α-tubulin and inactivation of the NLRP3 inflammasome. This mechanism contributes to the amelioration of acute kidney injury (AKI) and myocardial injury induced by sepsis (20, 21). However, recent studies have demonstrated that KAT2A also possesses succinyltransferase activity and exhibits higher affinity for succinyl-CoA than for acetyl-CoA (22, 23). However, it is regrettable that no studies have yet elucidated how KAT2A-mediated Ksucc specifically influences the pathogenesis and progression of sepsis. SIRT5 is the most extensively studied desuccinylase, primarily localized in mitochondria. It exhibits NAD+-dependent desuccinylation activity, which reduces the succinylation levels of mitochondrial proteins, thereby modulating the target activity of substrate proteins to maintain metabolic homeostasis (15, 24–26). SIRT7 is predominantly found in the nucleus, where it performs essential functions such as stimulating ribosomal RNA expression, facilitating DNA damage repair, and regulating chromatin compaction (27, 28). Studies have demonstrated that pyruvate kinase M2 (PKM2) serves as a critical target of Ksucc in macrophages. SIRT5-mediated desuccinylation activates PKM2, thereby suppressing lipopolysaccharide (LPS)-induced interleukin-1β (IL-1β) production and preventing dextran sulfate sodium (DSS)-induced colitis in mice (29). Wang et al. established an *in vitro* model of septic lung injury by stimulating lung epithelial cells with LPS. Their study demonstrated that SIRT5-mediated desuccinylation of Homeobox A5 (HOXA5) mitigated ferroptosis by activating ferroptosis suppressor protein 1 (FSP1), ultimately reducing LPS-induced lung injury (30). As a central kinase, TBK1 plays a pivotal role in both NF-κB and interferon regulatory factor (IRF) signaling pathways (13). The expression of pro-inflammatory cytokines and chemokines, including Interleukin-6 (IL-6), Tumor Necrosis Factor-α (TNF-α), and interferon-β (IFN-β), is contingent upon the activation of NF-kB and IRF3 (31). Research indicates that in endotoxemia and sepsis models, a reduction in macrophage SIRT5 levels leads to increased succinylation of TBK1, thereby impairing its interaction with IRF3 and TRAF2, which consequently suppresses the inflammatory response (13). Additionally, glutamine has been shown to enhance the activity of pyruvate dehydrogenase (PDH) in macrophages. This effect occurs through the inhibition of SIRT5-mediated desuccinylation of pyruvate dehydrogenase (PDHA1), leading to the restoration of PDH activity. This process promotes M2 polarization of macrophages and ameliorates burn sepsis in murine models (32). In summary, succinylation has been demonstrated to be a naturally occurring lysine modification that participates in nearly all biological processes within organisms. It plays a critical role in the initiation and progression of sepsis, as well as the associated organ dysfunction, suggesting that the regulation of Ksucc could serve as a promising therapeutic target for sepsis.

## S-palmitoylation

Palmitoylation is a PTM in which palmitic acid or palmitoyl-CoA acts as the acyl donor. This modification involves the covalent binding of palmitic acid to the amino acid residues of lysine, serine, glycine, threonine, and cysteine (**Figure 1**, **Table 1**) (33, 34). Based on the chemical linkage between fatty acids and proteins, palmitoylation modifications can be classified into S-palmitoylation, N-palmitoylation, and O-palmitoylation. Among these, S-palmitoylation has been the most extensively studied (10). Palmitoylation occurs through both enzymatic and non-enzymatic mechanisms, with the enzymatic process being the dominant form of catalysis (6). Palmitoylation is catalyzed by palmitoyltransferases (PATs) that contain the zinc finger Asp-His-His-Cys (ZDHHC) motif, whereas depalmitoylation is mediated by acyl-protein thioesterases (APTs) (35–37). S-Palmitoylation, predominantly observed in eukaryotic cells, modulates the membrane affinity of substrate proteins, thereby influencing their stability, subcellular localization, and interactions with other proteins. This post-translational modification has been implicated in various pathological conditions, including cancer, inflammation, and cardiovascular diseases (38–41). Studies have demonstrated that under inflammatory conditions, both palmitoylation and DHHC21 activity are significantly upregulated (42, 43). Palmitoylation serves as a novel regulatory factor for the formation and function of extracellular vesicles (EVs) during sepsis. EVs isolated from wild-type septic mice significantly promoted neutrophil adhesion, migration, and the formation of neutrophil extracellular traps (NETs). However, DHHC21 deficiency inhibited this process by blocking palmitoylation, thereby reducing neutrophil infiltration and improving survival rates in mice (44). In addition, the NOD-like receptor family pyrin domain-containing 3 (NLRP3) plays a critical role in the pathogenesis of sepsis, with inflammatory pathways being tightly regulated through dynamic palmitoylation of NLRP3. Multiple studies have demonstrated that inhibition of the NLRP3 inflammasome can ameliorate sepsis-induced inflammation and associated organ dysfunction (45–48). Studies have demonstrated that vaccarin inactivates the NLRP3 inflammasome by promoting its palmitoylation via zDHHC12, thereby ameliorating LPS-induced cardiomyopathy (49). Furthermore, zDHHC12-mediated palmitoylation facilitates NLRP3 degradation via the chaperone-mediated autophagy pathway. The absence of zDHHC12 exacerbates inflammatory symptoms and increases mortality in LPS-induced endotoxic shock (50, 51). The NLRP3 inflammasome triggers and amplifies inflammatory responses by inducing pyroptosis and the secretion of inflammatory cytokines, a process that is dependent on gasdermin D (GSDMD) (52). Numerous studies have demonstrated that upon LPS stimulation of macrophages, GSDMD undergoes palmitoylation modification at Cys191/192. Moreover, the upregulation of LPS-induced DHHC7 autopalmitoylation has been shown to play a critical role in GSDMD palmitoylation (53–56). Therefore, LPS may induce GSDMD palmitoylation by upregulating the expression levels of DHHC palmitoyltransferases and their autopalmitoylation activity (57), Inhibition of inflammasome-stimulated GSDMD palmitoylation significantly suppresses GSDMD-dependent pyroptosis and IL-1β release, mitigates organ injury, and improves survival rates in septic mice (55). Palmitoylation of GSDMD-NT may serve as a novel therapeutic target for sepsis. In addition to the aforementioned mechanisms, palmitoylation serves as a crucial post-translational modification in the regulation of the STING pathway (58). In sepsis, stimulator of interferon genes (STING) is hyperactivated and phosphorylated, leading to excessive production of inflammatory cytokines such as IL-6, IFN-β, and TNF-α (59). Moreover, the activation of STING in platelets serves as a critical driver of sepsis-induced pathological alterations (60). Studies have demonstrated that fatty acid synthase (FASN) suppresses STING palmitoylation via malonyl-CoA, thereby alleviating sepsis-induced liver injury (61). In summary, S-palmitoylation is essential in the pathogenesis of sepsis and its induced multi-organ dysfunction. A deeper understanding of the molecular mechanisms underlying S-palmitoylation will provide novel perspectives on the pathophysiological processes of sepsis and identify more accurate therapeutic targets for future interventions.

## Lactylation (Kla)

Lactate, the end product of glycolysis, was historically regarded as a metabolic waste product until 2019, when the research team led by Yingming Zhao discovered that lactate serves as the precursor for histone lysine lactylation (Kla) (**Figure 1**, **Table 1**) (62). Current evidence indicates that lactate has emerged as an epigenetic modification substrate capable of inducing lactylation on lysine residues of both histones and non-histone proteins, thereby playing a regulatory role in gene transcription and protein function (63–65). Lactylation modification is closely associated with inflammation and plays a pivotal role in regulating inflammatory responses (66). The lactylation of histones H3 and H4 is a p53-dependent process mediated by the acetyltransferase p300, which influences transcriptional regulation of genes (62). Clinical study results demonstrated that compared with the healthy control group, septic shock patients exhibited significantly elevated levels of lactosylation and H3K18la expression. Furthermore, H3K18la expression was positively correlated with IL-6, Acute Physiology and Chronic Health Evaluation II (APACHE II) score, and Sequential Organ Failure Assessment (SOFA) score (67, 68). Lactate Promotes LPS-Induced NF-κB Signaling Pathway Activation in Bovine Mammary Epithelial Cells via p300/CBP-Mediated H3K18 Lactylation (69). Moreover, lactate can upregulate heparanase in pulmonary microvascular endothelial cells through histone H3K18 lactylation, thereby promoting degradation of the pulmonary endothelial glycocalyx and exacerbating sepsis-associated acute lung injury (70). Moreover, histone lactylation plays a pivotal role in macrophage polarization. The gradual accumulation of lactate in macrophages results in a significant increase in histone lactylation, thereby promoting the transition of macrophages from the M1 to the M2 phenotype (62, 71). Subsequent studies have revealed that lactylation modification is not restricted to histones but also occurs on non-histone proteins. High mobility group box 1 (HMGB1), a highly conserved and ubiquitously expressed protein, is released by activated macrophages and plays a critical role in inducing innate immune responses and amplifying proinflammatory signaling pathways during sepsis (72). The regulation of HMGB1 lactylation by p300/CBP plays a crucial role in the progression of sepsis. In polymicrobial sepsis, the acetyltransferase p300/CBP acts as a key enzyme that promotes HMGB1 lactylation in macrophages. Lactylated/acetylated HMGB1 is secreted through exosomes, where it disrupts endothelial cell integrity and impairs endothelial barrier function (73). Moreover, HMGB1 lactylation induced by lactate accumulation can activate the HMGB1-NETs signaling pathway, thereby contributing to and worsening mouse SAKI (74, 75). These findings provide strong support for HMGB1 as a therapeutic target in the treatment of sepsis and its associated organ dysfunction. Elevated serum lactate concentration is closely associated with increased mortality in patients with sepsis (66). Research indicates that both endogenous and exogenous increases in lactate levels exacerbate SAKI by mediating the lactylation of fission 1 (Fis1). Conversely, reducing lactate levels and Fis1 lactylation can alleviate AKI (76).This study uncovers a novel mechanism connecting lactate levels to organ damage in sepsis, emphasizing the critical importance of therapeutic strategies focused on reducing serum lactate levels in sepsis patients. Overall, lactate and its associated lactylation modifications play a crucial role in the pathogenesis and progression of sepsis. Further investigation into the molecular mechanisms underlying lactylation will offer new perspectives for understanding the pathological processes of sepsis and provide more precise therapeutic targets. As scientific research continues to advance, we anticipate uncovering more complex interactions between lactylation modifications and sepsis.

## Crotonylation (Kcr)

Crotonylation (Kcr) is a short-chain lysine acylation modification, in which a crotonyl group is transferred to a lysine residue under the action of crotonyltransferases using crotonyl-CoA as the substrate (**Figure 1**, **Table 1**) (77). While many regulatory factors and sites overlap between Kcr and Kac, the distinctive planar structure and four-carbon chain length of Kcr differentiate it from Kac (22). Crotonyltransferases and de-crotonylases dynamically regulate histone crotonylation. Additionally, various factors, including the levels of crotonyl-CoA and the activities of positive regulators [acyl-CoA oxidase 1 (ACOX1), acyl-CoA oxidase 3 (ACOX3), acyl-CoA dehydrogenase short-chain (ACADS), and acyl-CoA synthetase short-chain family member 2 (ACSS2)] and negative regulators [chromodomain Y-like (CDYL) and short-chain enoyl-CoA hydratase 1 (ECHS1)], govern the Kcr modification of proteins (77, 78). Lysine crotonylation plays a crucial role in bacterial infections. A study found that following methicillin-resistant Staphylococcus aureus (MRSA) infection in THP1 cells, the crotonylation modification profile was altered, with 1,384 downregulated crotonylation sites across 899 proteins and 193 upregulated crotonylation sites across 160 proteins (79). Research has demonstrated that supplementation with crotonyl-CoA in macrophages results in a substantial increase in histone H3K18Cr. Additionally, knockdown of ACSS2, an enzyme involved in crotonyl-CoA synthesis, reduced histone H3K18Cr levels and the expression of inflammatory genes in LPS-stimulated RAW264.7 cells. These findings indicate a strong link between crotonylation and sepsis, suggesting that modulation of crotonylation could regulate the progression of inflammation (80). Furthermore, short-chain fatty acids (SCFAs) can modulate sepsis progression by regulating lysine crotonylation modifications through the inhibition of histone deacetylase (HDAC). Therefore, elucidating the mechanistic roles of both histone and non-histone Kcr modification targets in sepsis pathogenesis is critical for developing effective therapeutic strategies (80). However, our understanding of the underlying mechanisms by which Kcr influences sepsis progression remains incomplete. Future studies should focus on elucidating the specific mechanisms through which Kcr modulates sepsis progression and its functional role in sepsis pathogenesis.

## 2-hydroxyisobutyrylation (Khib)

Lysine 2-hydroxyisobutyrylation (Khib), a novel acylation modification first identified in 2014, utilizes 2-hydroxyisobutyric acid and its coenzyme-acylated form, 2-hydroxyisobutyryl-CoA, as substrates (**Figure 1**, **Table 1**). This modification is conserved in both eukaryotic and prokaryotic cells and is involved in a variety of biological processes (81–83). The hallmark of Khib is the addition of a hydroxyl group to lysine residues (7). These PTMs not only affect histones but also regulate non-histone proteins, participating in diverse physiological processes including glycolysis, gluconeogenesis, and the TCA cycle. Khib plays a crucial role in modulating both normal physiological functions and pathological developments in mammals (81, 84). The acetyltransferase Tip60 has been identified as a “writer” of Khib in mammalian cells, demonstrating catalytic activity for Khib modification both ex vivo and *in vivo* (85). Furthermore, the p300 protein has been shown to catalyze Khib, while HDAC1-3 and sirtuin 3 (SIRT3) exhibit de-2-hydroxyisobutyrylase activity (83, 84). Currently, research on the relationship between Khib and sepsis is limited. However, it is noteworthy that 2-hydroxyisobutyric acid, a SCFA, is significantly upregulated in various inflammatory diseases. This suggests that alterations in its levels, along with Khib, are closely associated with the onset, progression, and regulation of inflammation (80). Research has indicated that in the skin tissues of psoriasis patients, proteins encoded by the S100A9, FUBP1, and SERPINB2 genes exhibit significant Khib modifications, which are associated with proteins in the PI3K-Akt signaling pathway (86). Notably, S100A9 is a potential therapeutic target for sepsis, though further investigation is required to determine whether its Khib modification plays a role in the onset and progression of sepsis (87). Although there is currently a lack of direct evidence linking Khib to sepsis, Khib is fundamentally involved in the regulation of various inflammation-related diseases. This underscores the need for further investigation into the role of Khib modifications in histones and non-histones in the onset and progression of sepsis, to identify potential new therapeutic targets for sepsis. In conclusion, as a novel epigenetic modification, Khib research is still in its early stages; however, its significant role in epigenetic regulation offers promising new directions for the early diagnosis and treatment of sepsis.

## β-hydroxybutyrylation (Kbhb)

Lysine β-hydroxybutyrylation (Kbhb) is a novel protein acylation modification mediated by β-hydroxybutyrate (**Figure 1**, **Table 1**) (88, 89). To date, Kbhb has been detected in Drosophila, yeast, murine, and human cells, with 46 identified Kbhb sites distributed across the four core histones (H2A, H2B, H3, and H4) and the linker histone H1 (90, 91). P300/CBP are well-known acyltransferases that catalyze the covalent attachment of β-hydroxybutyryl-CoA to lysine residues, resulting in the formation of Kbhb (91). HDAC1-3 and SIRT1-3 have been identified as de-β-hydroxybutyrylases, which remove Kbhb modifications, thus maintaining the homeostasis of intracellular modification levels (90, 92). Notably, in contrast to HDAC1-3, SIRT3 exhibits class-selective histone de-β-hydroxybutyrylase activity (91, 93, 94). Additionally, 3-hydroxy-3-methylglutaryl-CoA synthase 2 (HMGCS2) and β-hydroxybutyrate dehydrogenase 1 (BDH1), key enzymes involved in ketone body metabolism, can indirectly modulate histone Kbhb modifications (90). Moreover, β-hydroxybutyrate, as a critical substrate, influences Kbhb in a concentration-dependent manner. β-Hydroxybutyrate (BHB) is the most abundant ketone body in human blood, accounting for approximately 70% of total ketones (95). Early clinical studies demonstrated that serum BHB levels were significantly higher in sepsis survivors compared to non-survivors (96). Mechanistically, β-HB suppresses the activation of NF-κB and the NLRP3 inflammasome, thereby exerting anti-inflammatory, anti-apoptotic, and mitochondrial dysfunction-antagonizing effects (97, 98). Additionally, it functions as a histone deacetylase inhibitor, contributing to antioxidative properties and the reduction of pro-inflammatory macrophage activation (99, 100). The ketogenic diet (KD), a high-fat, low-carbohydrate, moderate-protein dietary regimen, has been utilized clinically since the 1920s (101). KD decreases glucose utilization and promotes the conversion of free fatty acids in the liver into ketone bodies (primarily BHB). A study in COVID-19 patients demonstrated that KD significantly reduced IL-6 levels in these individuals (102). Furthermore, a single-center study evaluating the efficacy and safety of the ketogenic diet in sepsis patients showed that ventilation-free, vasopressor-free, dialysis-free, and intensive care unit–free days were higher in the ketogenic group (103). Unfortunately, these studies did not examine the impact of BHB on Kbhb, which complicates the understanding of how β-hydroxybutyrate affects sepsis through Kbhb modifications. Mass spectrometry analysis has revealed that Kbhb modifications also occur in non-histone proteins, especially in transcription factors and key metabolic enzymes. Furthermore, β-hydroxybutyrate may influence disease progression by modulating non-histone Kbhb (98, 104). It is noteworthy that elevated ketone body levels in septic patients may increase the risk of fatal metabolic acidosis due to impaired energy metabolism and mitochondrial dysfunction. A dose-exploration study of ketone salts demonstrated that a dosage of 40 mmol/kg/day approaches the toxicity threshold. In fact, a 20% daily dose escalation, due to excessive Na^+^ intake, exacerbates organ damage and increases mortality (105, 106). Therefore, further research is required to establish the safety of elevating ketone body levels in the treatment of septic patients. This indicates that regulating the Kbhb levels of key metabolic enzymes and critical genes (via ketogenic diets, fasting, or direct supplementation of β-hydroxybutyrate) could potentially slow the progression of sepsis. In conclusion, Kbhb connects metabolic reprogramming with epigenetic regulation, highlighting its significant biological functions. This provides new avenues for exploring chromatin regulation and the roles of β-hydroxybutyrate in both physiological and pathological processes. Consequently, Kbhb represents a promising clinical target with vast research potential, especially in sepsis and associated organ dysfunction, and is poised to become a new focal point in research.

## Malonylation (Kmal)

Lysine malonylation (Kmal) is a novel acylation modification based on malonyl-CoA that was first identified in bacteria and mammals in 2011 and is involved in various pathophysiological processes (**Figure 1**, **Table 1**) (107). Kmal is regulated by the concentration of malonyl-CoA, which is modulated by acetyl-CoA carboxylase (ACC), propionyl-CoA carboxylase (PCC), fatty acid synthase (FASN), and malonyl-CoA decarboxylase (MCD) (6). Additionally, similar to other acylation modifications, Kmal is controlled by acyltransferases and demodification enzymes. Studies have demonstrated that acetyltransferase 2A (KAT2A) can function as a lysine malonyltransferase. In animal models, knockdown of KAT2A specifically reduces histone Kmal levels (108), while SIRT5 exhibits significant demalonylase activity (109). Kmal is highly prevalent in mitochondrial proteins involved in regulating metabolic pathways, including glycolysis, fatty acid oxidation, and inflammation, particularly in endothelial cells and macrophages. Macrophages typically undergo two polarized states—classical activation (M1 type) and alternative activation (M2 type)—each serving distinct functions in inflammatory responses and tissue homeostasis. LPS stimulation can influence the Kmal levels of various proteins (110). Studies indicate that in LPS-stimulated macrophages, elevated malonyl-CoA levels upregulate Kmal, thereby modulating inflammatory signaling and enhancing the production of pro-inflammatory factors. The mechanism involves Kmal modification of lysine at position 213 in GAPDH, resulting in increased enzyme activity and dissociation from TNF-α mRNA, which subsequently promotes TNF-α translation (110). Additionally, in LPS-induced RAW264.7 cells, Atractylodin has been shown to inhibit GAPDH Kmal, leading to a reduction in TNF-α levels (111). In conclusion, Kmal modification plays a pivotal role in the regulation of sepsis. Future research should focus on identifying other “write-erase” enzymes involved in this modification, pinpointing their specific or non-specific sites, and assessing the Kmal levels of histones and non-histones relevant to sepsis. Ultimately, further investigation into Kmal will provide novel insights and approaches for the diagnosis and treatment of sepsis.

## Crosstalk between metabolism and novel protein acylation in sepsis

**Figure 2:**
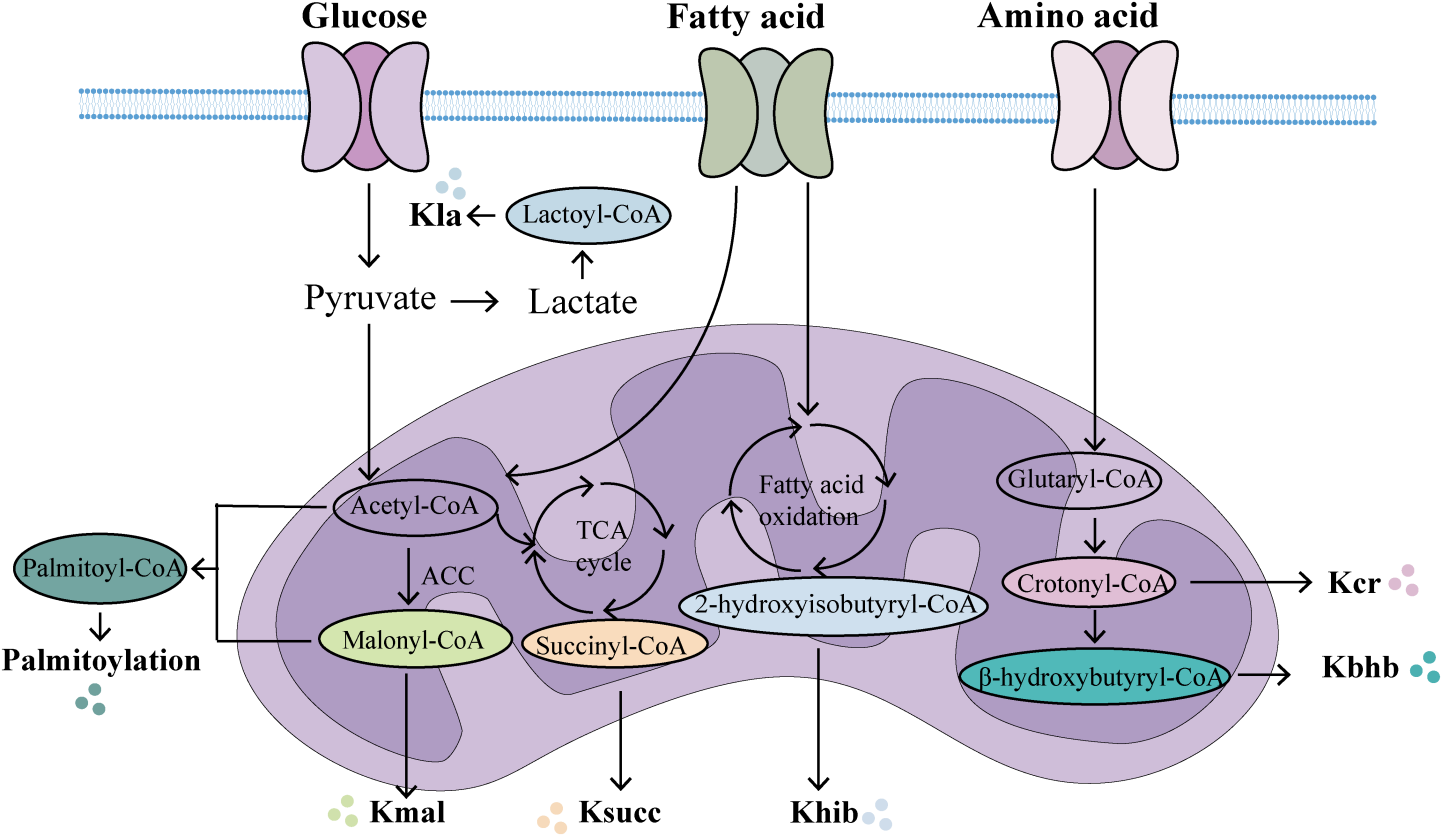
The main metabolic origins of novel acylations. In the mitochondria, multiple metabolic pathways generate acyl-CoA, which provides acylation groups to covalently modify proteins. (Created with BioGDP.com). Kla, lactylation; Kcr, crotonylation; Kbhb, β-hydroxybutyrylation; Khib, 2-hydroxyisobutyrylation; Ksucc, succinylation; Kmal, malonylation.

PTMs have emerged as pivotal regulators in shaping protein functions, providing novel insights into the intricate interplay between metabolic and phenotypic regulation in sepsis. Metabolites can modulate protein acylation by serving as acyl group donors or by altering the activity of acyltransferases and deacylases. Conversely, protein acylation participates in critical cellular processes associated with both physiology and pathology, including protein stability, subcellular localization, enzymatic activity, transcriptional regulation, protein-protein interactions, and protein-DNA interactions (112). Acyl-CoA acts as an acyl group donor in protein acylation modifications, primarily sourced from metabolic products of glucose, fatty acids, and amino acids (**Figure 2**). For example, lactate, a product of glycolysis and a key energy source, plays a crucial role in lactylation modification. Additionally, lipid β-oxidation increases the abundance of crotonyl-CoA, β-hydroxybutyryl-CoA, and palmitoyl-CoA, thereby facilitating the corresponding modifications. In addition, amino acid catabolism significantly contributes to acetylation and crotonylation via acetyl-CoA and crotonyl-CoA. The levels of protein acylation are closely associated with intracellular acyl-CoA concentrations and are thus dynamically regulated by metabolic status (113). In sepsis, the activation of inflammatory factors such as IL-1, IL-6, and TNF-α can directly or indirectly mediate metabolic alterations (114). Additionally, during the acute phase of sepsis, extensive immune system activation drives a shift in energy metabolism from oxidative phosphorylation and fatty acid β-oxidation to glycolysis, a process commonly referred to as the Warburg effect (115). PKM2, a critical enzyme in glycolysis, has been shown to reduce lactate production and cytokine release both *in vitro* and *in vivo* upon knockdown, thereby protecting mice from fatal endotoxemia and sepsis-related damage (116). During sepsis, significant disruptions occur not only in glucose metabolism but also in lipid and amino acid metabolism, resulting in changes to cellular metabolite levels and activity. The modification of these metabolites can influence the pathophysiological processes of sepsis by modulating enzyme activity or altering the structure and interactions of proteins (73). Notably, acylation modifications can take place in histones, affecting gene expression regulation, or in non-histones, impacting their function or protein-protein interactions (117, 118). Thus, alterations in metabolite levels during sepsis can modulate inflammation and immune responses by regulating the levels of acylation modifications. In summary, nearly all types of acylation modifications correspond to metabolic changes. The metabolic reprogramming and shifts in the external metabolic microenvironment during sepsis can regulate protein acylation. Conversely, protein acylation modifications can also reprogram and influence the metabolic state (112).In summary, future research should further explore metabolic reprogramming and protein acylation modifications, offering deeper insights into the interrelationship between metabolic reprogramming, acylation modifications, and sepsis.

## Summary and perspectives

Sepsis is a life-threatening condition triggered by infection, leading to a systemic inflammatory response syndrome and often resulting in multiple organ dysfunction (119, 120). Current treatment strategies emphasize comprehensive interventions, including antibiotic therapy, fluid resuscitation, hemodynamic support, immune modulation, and organ support (121, 122). However, due to the complex pathophysiology and high heterogeneity of sepsis, no single treatment has been proven effective in improving patient survival. As a result, the exploration of new therapeutic approaches is essential. Recently, novel acylation modifications have attracted significant attention as promising therapeutic targets. This review highlights recent advances in protein acylation research, focusing on the properties and functions of seven novel acylation modifications and their potential relevance in sepsis and sepsis-induced multiple organ dysfunction.

Currently, several studies have extensively investigated novel pharmacological agents targeting sepsis-induced acylation modifications. Research has demonstrated that myeloid differentiation primary response 88 (MYD88) serves as the most prevalent adaptor molecule within the Toll-like receptor (TLR) family. Inhibition of MYD88 S-palmitoylation has been shown to suppress TLR-mediated inflammatory responses (123). 2-bromopalmitic acid (2BP) is a commonly used irreversible inhibitor of palmitoylation that suppresses TLR2 S-palmitoylation, resulting in reduced TLR2 expression on the plasma membrane and attenuated inflammatory responses (124). However, due to its toxicity and off-target effects, including interference with fatty acid metabolism, its clinical application is not feasible (125). Furthermore, selectively manipulating the palmitoylation status of specific target proteins may offer a novel approach to mitigate adverse effects (126). The clinical application of the depalmitoylation inhibitor Palm-B is also limited due to its inhibitory effect on the activity of a series of serine hydrolases (127). A feasible strategy for drug development involves screening compound libraries. In a recent study, screening of a covalent compound library containing 565 compounds identified NU6300 as capable of covalently reacting with C191 of GSDMD. This interaction blocks GSDMD cleavage and palmitoylation, thereby inhibiting GSDMD-N membrane translocation and subsequent oligomerization. The compound effectively suppressed pyroptosis both *in vivo* and ex vivo, while mitigating inflammatory responses (128). Metabolic reprogramming represents one of the hallmark characteristics of sepsis. Metabolic intermediates can directly drive novel acylation modifications, modulating chromatin structure and gene transcription, thereby forming intricate and dynamic feedback loops with epigenetic regulation. These metabolism-driven PTMs not only demonstrate the direct substrate role of metabolites but also highlight the bidirectional regulatory relationship between metabolism and epigenetic processes. Therefore, alongside pharmacological treatments, dietary strategies such as ketogenic diets and restrictions on glucose or long-chain fatty acid intake may prevent or treat sepsis by modulating protein acylation through metabolic intermediates. However, the optimal concentration of BHB that can be employed without inducing lethal acidosis remains unclear. Carefully designed clinical and preclinical studies are required to achieve a comprehensive understanding of the molecular basis of BHB in epigenetics and other biological processes, particularly considering its interplay with other acylation modifications and potential side effects induced by ketosis-promoting therapeutic interventions. With advancing understanding of novel acylation modifications in sepsis, it is warranted to anticipate that further studies will be conducted to explore effective therapeutic strategies for sepsis management.

Although PTMs hold significant potential as novel therapeutic targets for sepsis, they currently face several challenges. Firstly, despite rapid advancements in mass spectrometry technology, detection sensitivity may remain insufficient for low-abundance proteins and amino acid residues with low modification stoichiometry (10). Additionally, certain detection methods may exhibit specificity issues, potentially leading to false-positive or false-negative identification of PTMs. Secondly, when multiple modifications occur on the same amino acid residue, potential interactions between different PTMs may arise, thereby increasing the complexity of PTM networks (129). Furthermore, PTMs typically occur rapidly and are difficult to quantify, making the investigation of their crosstalk particularly challenging (130). Thirdly, as PTMs predominantly occur intracellularly, direct targeting of specific modifications through external drug administration remains technically demanding. Even if intracellular drug delivery is achieved, ensuring precise targeting of the intended PTMs presents a significant pharmacological challenge (131). In conclusion, continuous development and refinement of detection technologies are required to overcome these limitations, thereby enabling a more comprehensive understanding of protein functions and mechanisms of action. Once these challenges are addressed, acylation modifications are expected to play a more significant role in the diagnosis and treatment of sepsis and its associated multiple organ dysfunction syndrome.

This review offering new insights into novel acylation modifications in the pathogenesis of the sepsis. Through a comprehensive investigation of the signaling pathways and molecular mechanisms that regulate PTMs, we aim to clarify their specific involvement in sepsis-induced organ dysfunction, providing a stronger theoretical basis for the development of targeted drugs and therapeutic strategies. This study has several limitations. First, a considerable proportion of existing studies have employed LPS-induced inflammatory models, either *in vitro* or *in vivo*, to investigate the functions of acylation modifications. While LPS stimulation effectively mimics the early hyperinflammatory phase of sepsis by activating TLR4-mediated signaling cascades, it fails to recapitulate the complex immunological dynamics observed in clinical sepsis. Second, the temporal and spatial dynamics of acylation modifications during the progression of sepsis remain largely unexplored. Large-scale clinical studies are needed to evaluate the diagnostic and prognostic value of these novel acylation modifications in sepsis.
